# Development and Antitumor Evaluation of Doxorubicin-Loaded Two-Layered Sheets for Local Chemotherapy via Direct Drug Application to the Tumor Surface

**DOI:** 10.3390/pharmaceutics17121565

**Published:** 2025-12-04

**Authors:** Nao Mitsudome, Mayuko Matsumoto, Yui Murakami, Fei Yuan, Hirotaka Miyamoto, Shintaro Fumoto, Akira Toriba, Koyo Nishida

**Affiliations:** Graduate School of Biomedical Sciences, Nagasaki University, 1-7-1 Sakamoto, Nagasaki 852-8501, Japan; bb55723005@ms.nagasaki-u.ac.jp (N.M.); hmiyamoto@nagasaki-u.ac.jp (H.M.); sfumoto@nagasaki-u.ac.jp (S.F.); toriba@nagasaki-u.ac.jp (A.T.)

**Keywords:** doxorubicin, sheet, additive, poly(lactic-*co*-glycolic acid), local administration, biodistribution

## Abstract

**Background/Objectives:** Non-specific distribution of anticancer drugs to non-lesional areas of the body and organs, followed by their rapid disappearance, limits the drug concentration in the tumor, impacting the chemotherapy efficacy. To address this issue, local drug administration is important for controlled drug distribution. Therefore, this study aimed to design two-layered sheets loaded with doxorubicin (DOX) and 10 or 20% (*w*/*w*) additives for direct tumor application. **Methods:** Polyethylene glycol, hydroxypropyl cellulose, and polyvinylpyrrolidone were used as additives to prepare poly(lactic-*co*-glycolic acid)-based DOX-loaded sheets. *In vitro* release, *in vivo* antitumor effects, and biodistribution of the drug were evaluated in mice. **Results:** Additive-containing sheets exhibited diverse *in vitro* release profiles and several sheets exerted significantly more potent antitumor effects than additive-free DOX sheets in Hepa1-6 tumor-bearing mice. Fourteen days after application, DOX was majorly distributed within the tumor, and its concentrations in most normal organs were below the detection limit. The overall antitumor effects of the additive-containing sheets, as measured by the area under the curve, were statistically indistinguishable; however, mean concentration in the 20% polyethylene glycol-DOX sheet group was approximately 7-fold higher than that in the 20% hydroxypropyl cellulose-DOX sheet group. These findings suggest that antitumor mechanisms do not rely solely on the absolute drug concentration at the tumor site. **Conclusions:** Overall, our findings provide important insights for the development of DOX-loaded two-layered sheets for direct tumor application.

## 1. Introduction

Cancer is the first or second leading cause of death among people under the age of 70 years in 112 countries, and its incidence and mortality rates are rapidly increasing worldwide [1]. With an estimated 28.4 million new cases of cancer worldwide by 2040, development of innovative treatment strategies is crucial for its control [1,2]. Chemotherapy remains one of the most widely used treatments for various cancers [3]. However, conventional chemotherapy can cause severe side effects, as anticancer drugs may be non-specifically distributed throughout the body and in non-lesional areas of organs. Additionally, rapid clearance limits the actual drug concentration delivered to tumors. All these pose major barriers to effective cancer chemotherapy [4,5,6,7].

Local drug administration is one approach to overcome these treatment challenges. Local administration controls the drug distribution process [8], limits the first-pass effect [9] and blood circulation, and increases the drug concentrations in target tissues [9]. Owing to these advantages, local chemotherapy is important for solid tumor treatment [10]. Many locally administered drug delivery systems have been developed for direct tumor application, such as via intratumoral insertion of implants or intratumoral injection of hydrogels; these systems have successfully delivered high drug concentrations to tumors and achieved sustained antitumor effects, while reducing toxicity in normal tissues [11] and drug distribution to normal organs [11,12]. We previously developed an organ surface drug administration system to achieve selective drug distribution via direct drug absorption on organ surfaces in the abdominal cavity, such as the liver [13]. Furthermore, we developed a two-layered sheet formulation applicable to organ surfaces and assessed its pharmacokinetic performance [14]. Application of such sheets to cancer lesion surfaces can enhance the use of anticancer drugs with serious side effects or stability issues by directly delivering drugs to the target sites. Doxorubicin (DOX) is widely used to treat various cancer types. However, cardiotoxicity is a common adverse effect of DOX [15], with the risk of cardiomyopathy increasing at cumulative doses >550 mg/m^2^ [16]. Although various polymeric DOX delivery systems, such as nanoparticles and micelles, have been developed to mitigate systemic toxicity [17,18,19], these systemic approaches often face challenges, including limited sustained tumor accumulation [20] and residual off-target toxicities [21]. In contrast, direct administration to the tumor surface can reduce DOX distribution to the heart by selectively delivering the drug to the target site, thereby improving its therapeutic efficacy.

In this study, DOX-loaded sheets were prepared as local DOX delivery systems. To control drug release, polyethylene glycol (PEG), hydroxypropyl cellulose (HPC), and polyvinylpyrrolidone (PVP) were used as additives at 10% or 20%. This study mainly aimed to directly apply the additive-containing DOX-loaded sheets to mouse tumors and evaluate their *in vivo* antitumor effects and biodistribution. To determine the factors contributing to the antitumor effects of the additive-containing DOX sheets, the associations between tumor DOX concentrations and overall antitumor effects were explored. Our results can aid in enhanced formulation design to improve the therapeutic efficacy of DOX-loaded sheets for localized chemotherapy.

## 2. Materials and Methods

### 2.1. Materials

DOX hydrochloride was purchased from Tokyo Chemical Industry Co., Ltd. (Tokyo, Japan). Poly(lactic-*co*-glycolic acid) (PLGA; lactide:glycolide = 65:35; Mw: 40,000–75,000) was purchased from Sigma-Aldrich (St. Louis, MO, USA). PEG 4000 was purchased from Nacalai Tesque, Inc. (Kyoto, Japan), and HPC (viscosity range: 2.0–2.9 mPa·s) and PVP K30 were purchased from FUJIFILM Wako Pure Chemical Corporation (Osaka, Japan). Mouse hepatoma-derived Hepa1-6 cells were purchased from the Cell Engineering Division of the RIKEN BioResource Research Center (Tsukuba, Ibaraki, Japan). For cell culture, penicillin–streptomycin solution (100×) was purchased from FUJIFILM Wako Pure Chemical Corporation, and Dulbecco’s modified Eagle’s medium (high glucose; GlutaMAX supplement, pyruvate) was purchased from Thermo Fisher Scientific (Waltham, MA, USA). All other chemicals were of analytical grade.

### 2.2. Preparation of DOX-Loaded Two-Layered Sheets

PLGA was dissolved in acetone (FUJIFILM Wako Pure Chemical Corporation, Osaka, Japan) to prepare a 10% (*w*/*v*) solution. A cylindrical glass diffusion cell (inner diameter: 18 mm) was attached to a stainless-steel tray covered with plastic wrap, and 400 µL of PLGA solution was added to the cylindrical glass diffusion cell and allowed to air dry overnight at room temperature. Additive solutions were prepared by dissolving 1% (*w*/*v*) PEG, HPC, or PVP in acetone and stirring overnight. To prepare the DOX solution, DOX was dissolved in dimethyl sulfoxide (FUJIFILM Wako Pure Chemical Corporation, Osaka, Japan) at a concentration of 100 mg/mL. PLGA (400 µL), DOX (9 µL), and additive (0, 899, or 2022 µL) solutions were stirred overnight, added to the diffusion cell, and air-dried. After drying, the sheets were cut into semicircles with a diameter of 12 mm to prepare the DOX-loaded sheets containing 0, 10%, and 20% (*w*/*w*) of the additive as drug-loaded layers. To prepare sheets consisting only of PLGA as drug-free layers, 400 µL of PLGA solution was added to the diffusion cell, air-dried at room temperature, and cut into semicircles with a diameter of 18 mm. Then, DOX-loaded two-layer sheets were prepared by bonding the drug-loaded layer and drug-free layer using Aron Alpha A “Sankyo” (Daiichi Sankyo Co., Ltd., Tokyo, Japan). DOX-loaded two-layered sheets containing 0% additive in the drug-loaded layer are hereafter referred to as additive-free DOX sheets. Due to the limited scalability of the preparation method, *in vivo* experiments were generally conducted with independently prepared batches, although in some cases, *in vivo* and *in vitro* studies were paired using sheets derived from the same batch.

**Scheme 1:**
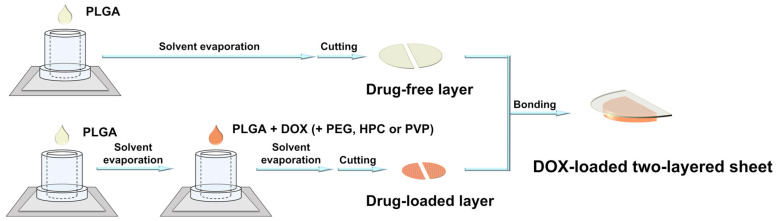
Schematic illustration of the preparation procedure for DOX-loaded two-layered sheets. Abbreviations: DOX, doxorubicin; PLGA, poly(lactic-*co*-glycolic acid); PEG, polyethylene glycol; HPC, hydroxypropyl cellulose; PVP, Polyvinylpyrrolidone.

The overall preparation procedure is illustrated in Scheme 1. The composition of all formulations at the preparation stage is summarized in Table 1.

### 2.3. Measurement of DOX-Loaded Two-Layered Sheet Thickness

The thickness of the additive-free sheets and the DOX-loaded sheets containing 10% or 20% additives (PEG, HPC, or PVP) was measured using a digital caliper (AS ONE Corporation, Osaka, Japan; resolution: 0.1 mm). Measurements were performed on three samples for each formulation (*n* = 3).

### 2.4. In Vitro Drug Release Studies

Each additive-free and -containing DOX-loaded sheet was floated individually in 20 mL of phosphate-buffered saline (PBS; pH 7.4), with the drug-loaded layer facing the water surface, and incubated with stirring at 200 rpm and 37 °C (EI-700V; AS ONE Corporation, Osaka, Japan). The sheets were transferred onto the surface of 20 mL of fresh PBS at each time point, and the media used for the release studies were collected over time.

### 2.5. Animals

All animal care and experimental procedures adhered to the Guidelines for Animal Experimentation of Nagasaki University and were approved by its Institutional Animal Care and Use Committee (approval no: 2004071622-4).

Five-week-old male Institute of Cancer Research (ICR) and C57BL/6J mice were purchased from Japan SLC, Inc. (Shizuoka, Japan). During rearing, food and water were provided ad libitum.

### 2.6. In Vivo Biodistribution Studies After DOX-Loaded Sheet Application to Subcutaneous Tissues

Five-week-old male ICR mice were randomly divided into seven groups as follows: (1) Additive-free DOX sheet, (2) 10% PEG-DOX sheet, (3) 20% PEG-DOX sheet, (4) 10% HPC-DOX sheet, (5) 20% HPC-DOX sheet, (6) 10% PVP-DOX sheet, and (7) 20% PVP-DOX sheet groups. From all mice (groups (1)–(7); *n* = 3/group), the abdominal skin and underlying subcutaneous tissues were incised after hair removal. Then, the sheets were applied to the subcutaneous tissues using Aron Alpha A “Sankyo.” The incision was sutured using silk sutures (Natsume Seisakusho Co., Ltd., Tokyo, Japan). One day after application, hair were removed, and the abdomen was opened to remove the subcutaneous tissues from the sheet-applied area. Blood was collected from the inferior vena cava using syringes pre-rinsed with heparin (Mochida Pharmaceutical Co., Ltd., Tokyo, Japan), and the liver, kidneys, spleen, lungs, and heart were removed. To quantify DOX concentrations in the excised subcutaneous tissues, organs, and plasma, wet weights of the excised subcutaneous tissues and organs were measured, and an extraction solution (a mixture of 1 M HCl and 2-propanol at a volume ratio of 1:1) was added, followed by homogenization to prepare homogenates. Blood was centrifuged at 10,000 rpm (approximately 8220× *g*) for 5 min using the MCX-150 centrifuge (TOMY Seiko Co., Ltd., Tokyo, Japan), plasma was collected, and the extraction solution was added. The homogenate solutions and plasma samples were incubated for 1 h at 4 °C and centrifuged at 3000 rpm (approximately 1670× *g* for the homogenate solutions and 825× *g* for the plasma samples) for 15 min at 4 °C using the RSL-IV centrifuge (Sakuma Seisakusho Co., Ltd., Tokyo, Japan) for the homogenates and Tabletop Micro Refrigerated Centrifuge 3500 (Kubota Corporation, Tokyo, Japan) for the plasma samples. The supernatants were collected and centrifuged again at 13,000× *g* for 15 min at 4 °C using the Kubota 3500 centrifuge.

### 2.7. Cell Lines and Animal Models

Hepa1-6 cells were cultured in the Dulbecco’s modified Eagle’s medium (high glucose, GlutaMAX supplement, pyruvate; Thermo Fisher Scientific) supplemented with 1% (*v*/*v*) penicillin–streptomycin solution (100× stock; FUJIFILM Wako Pure Chemical Corporation) and 10% (*v*/*v*) fetal bovine serum at 37 °C in a 5% CO_2_ environment. The abdomen was shaved, and 5 × 10^6^ cells suspended in 50 µL of PBS were subcutaneously injected into the left flank of five-week-old male C57BL/6J mice. Seven days after cell injection, ectopic subcutaneous tumor-bearing mice were generated.

### 2.8. Biodistribution and In Vivo Antitumor Effects of Additive-Containing DOX-Loaded Sheets

The seventh day after Hepa1-6 cell injection was set as day 0, and tumor-bearing male C57BL/6J mice were randomly divided into eight groups: (1) PLGA sheet, (2) additive-free DOX sheet, (3) 10% PEG-DOX sheet, (4) 20% PEG-DOX sheet, (5) 10% HPC-DOX sheet, (6) 20% HPC-DOX sheet, (7) 10% PVP-DOX sheet, and (8) 20% PVP-DOX sheet groups. The abdominal skin and underlying subcutaneous tissues were incised, and the sheets were applied to the tumor site using Aron Alpha A “Sankyo.” The incision was sutured using silk sutures. Tumor diameters in mice of the eight groups were measured at designated time points using a digital caliper (AS ONE Corporation, Osaka, Japan).

Tumor volume was calculated using Equation (1):

(1) $$Tumor\;volume\;({mm}^{3})={0.5\times a\;\left(mm\right)\times b}^{2}({mm}^{2})$$

where *a* and *b* represent the major and minor diameters, respectively.

Relative tumor volume was calculated by dividing the tumor volume at each time point by that on day 0; each group was analyzed using 3–6 mice. Then, area under the relative tumor volume–time curve over 14 days of application was calculated using the trapezoidal rule with Equation (2):

(2) $$Area\;under\;the\;relative\;tumor\;volume-time\;curve=\sum_{i=0}^{6}\frac{1}{2}\left(v_{i}+v_{i+1}\right)(t_{i+1}-t_{i})$$

where $v_{i}$ indicates the relative tumor volume at time *t_i_* (*t*_0_ = 0 d, *t*_1_ = 1 d, *t*_2_ = 3 d, *t*_3_ = 5 d, *t*_4_ = 7 d, *t*_5_ = 10 d, *t*_6_ = 12 d, and *t*_7_ = 14 d).

Mouse body weights in the additive-containing sheet groups (groups (3)–(8)) were measured at predetermined time points. Relative body weight was calculated by dividing the body weight at each time point by that on day 0, and 3–6 mice were evaluated in each group.

Tumor, blood, liver, kidney, spleen, lung, and heart samples were collected 1 and 14 days after application (*n* = 3/group at each time point) from groups (4) and (6) and 14 days after application (*n* = 3/group) from groups (3), (5), (7), and (8) for analysis. Blood was collected using syringes pre-rinsed with heparin. To quantify DOX concentrations in the excised tumors, organs, and plasma, supernatant samples of C57BL/6J mice were obtained using the same method used for ICR mice. The number of mice used to calculate the mean tumor concentration on day 14 and mean area under the relative tumor volume–time curve over 14 days differed among groups (3)–(5) due to differences in data availability, whereas the same mice were used for both calculations in groups (6)–(8).

### 2.9. DOX Quantification Method

To quantify DOX concentrations, fluorescence intensity of DOX was measured in each release medium used for *in vitro* studies and each supernatant used for *in vivo* studies with a spectrofluorophotometer (RF-6000; Shimadzu Corporation, Kyoto, Japan) at excitation and emission wavelengths of 500 and 550 nm, respectively. Plasma concentrations were assumed to have a density of 1 g/mL to facilitate direct comparisons with tissue concentrations.

Detection and quantification limits were calculated using Equations (3) and (4), respectively:

(3) $$Detection\;limit=3.3\times \frac{\sigma }{S}$$

(4) $$Quantification\;limit=10\times \frac{\sigma }{S}$$

where *σ* indicates the standard deviation of blank measurements, and *S* indicates the slope of the calibration curve.

### 2.10. Detection of DOX

Values below the detection limit were replaced with zero for calculation. When all three samples of each organ type from individual mice were below the detection limit, the values were presented as not detected. Values between the limit of detection and limit of quantification were considered semi-quantitative and included in the analysis as numerical values.

### 2.11. Statistical Analyses

Mean values, standard deviations for *in vitro* studies, and standard errors of the mean for *in vivo* studies were calculated for each group. Statistical tests were conducted using the R software (version 4.5.1; R Foundation for Statistical Computing, Vienna, Austria). Differences in *in vitro* release profiles and *in vivo* antitumor effects among groups were analyzed via two-way repeated-measures analysis of variance (ANOVA), followed by post hoc pairwise Welch’s *t*-tests with Bonferroni correction for multiple comparisons after assessment of variance homogeneity via Levene’s test using the car package (version 3.1-3). Relative body weight changes were analyzed via two-way repeated-measures ANOVA, followed by post hoc paired *t*-tests with Bonferroni correction to compare day 0 results with all subsequent time point results for each treatment group. Moreover, area under the relative tumor volume–time curve was compared among groups via one-way ANOVA. To assess biodistribution, two-way ANOVA, followed by paired *t*-tests with Bonferroni correction, was used for multiple comparisons between the application site and other organs and plasma samples. To compare each formulation to the control group (additive-free DOX sheets) in each tissue and plasma, Dunnett’s test was performed with the emmeans package (version 1.11.2). Additionally, homogeneity of variance was assessed using an F-test to compare two time points (days 1 and 14). An unpaired two-tailed Student’s *t*-test was used if the variances were not significantly different (*p* ≥ 0.05), whereas Welch’s *t*-test was applied if the variances were significantly different (*p <* 0.05). Effect sizes (Cohen’s *d*) and 95% confidence intervals (95% CIs) were calculated to assess the magnitude and precision of the observed differences. Cohen’s *d* was calculated using the pooled standard deviations for independent comparisons and the standard deviations of the mean difference for paired comparisons. Statistical significance was set at *p* < 0.05.

## 3. Results

### 3.1. In Vitro Evaluation of DOX-Loaded Sheets

#### 3.1.1. DOX-Loaded Two-Layered Sheet Thickness

The thickness of the prepared DOX-loaded two-layered sheets, both additive-free and additive-containing formulations, was measured using a digital caliper. Across the seven formulations, the mean sheet thickness ranged from 0.2 to 0.3 mm. Detailed thickness data for each sheet are provided in Supplementary Table S1.

#### 3.1.2. *In Vitro* Cumulative DOX Release Profiles

**Figure 1:**
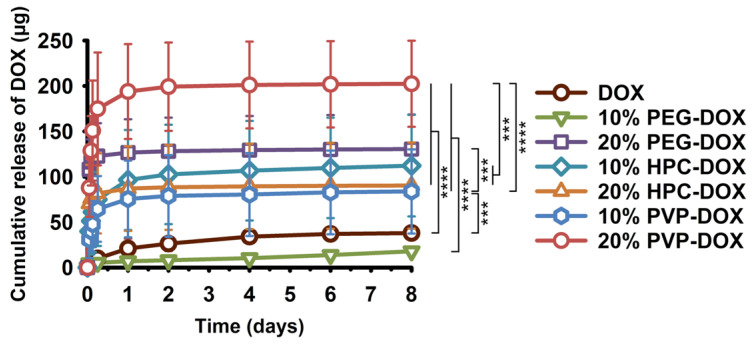
*In vitro* cumulative release profiles of doxorubicin (DOX) from additive-free DOX and DOX-loaded sheets containing 10 and 20% (*w*/*w*) polyethylene glycol (PEG), hydroxypropyl cellulose (HPC), and polyvinylpyrrolidone (PVP). Each point represents the mean ± standard deviation (SD) (*n* = 3). Asterisks indicate the significant differences between two groups, as determined via pairwise Welch’s *t*-test followed by Bonferroni correction (*** *p* < 0.001 and **** *p* < 0.0001).

To evaluate the effects of additive type and concentration on DOX release from the sheets, DOX sheets containing 10 or 20% additives (PEG, HPC, and PVP) were prepared, and their *in vitro* drug release profiles were evaluated. Additive-containing DOX sheets exhibited the fastest initial burst release rate for up to 1 h, followed by a gradual release. Compared to the additive-free DOX sheets, additive-containing DOX sheets showed significantly higher overall mean release, except for the 10% PEG-DOX sheets (Figure 1; *p* < 0.001; Cohen’s *d* range = 1.34–2.64; Supplementary Table S2). The different compositions of the additive-containing DOX sheets altered their release profiles, with the 10% PEG-DOX sheets showing significantly lower release than all the other additive-containing sheets.

### 3.2. In Vivo Biodistribution of DOX 1 Day After Application of DOX-Loaded Sheets to Mouse Subcutaneous Tissues

**Figure 2:**
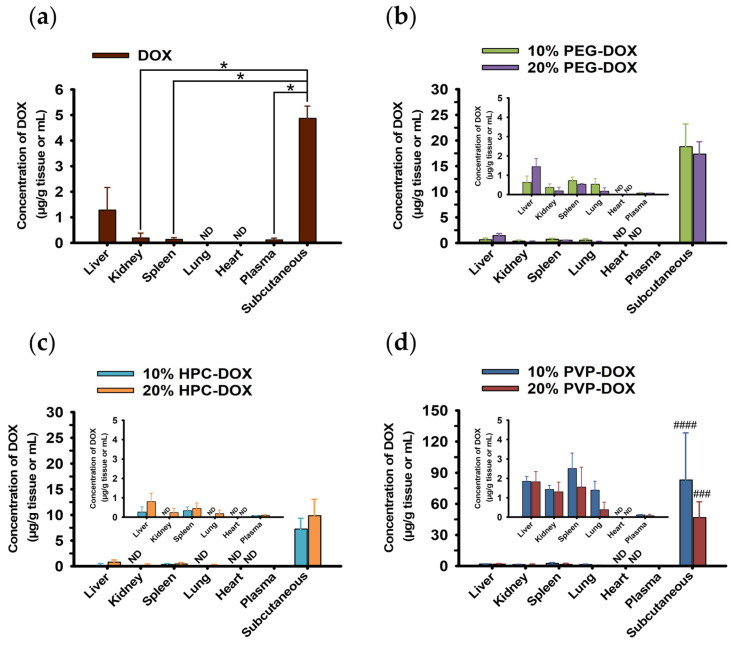
*In vivo* biodistribution of DOX 1 day after application of the following DOX-loaded sheets to Institute of Cancer Research (ICR) mouse subcutaneous tissues: (**a**) Additive-free DOX sheets, (**b**) 10 and 20% PEG-DOX sheets, (**c**) 10 and 20% HPC-DOX sheets, and (**d**) 10 and 20% PVP-DOX sheets. Insets in (**b**–**d**) show DOX concentrations in the major organs and plasma. Each bar represents the mean ± standard error of the mean (SEM) (*n* = 3). Asterisks indicate the significant differences between the subcutaneous tissues and other organs or plasma, as determined via paired *t*-tests followed by Bonferroni correction (* *p* < 0.05). Plasma concentrations were assumed to have a density of 1 g/mL for comparisons with tissue concentrations. Hash marks indicate the significant differences from the control group (additive-free DOX sheets), as determined via two-way analysis of variance (ANOVA) followed by Dunnett’s post hoc test (### *p* < 0.001 and #### *p* < 0.0001). Not detected (ND) indicates that all three samples from each organ type of each mouse were below the limit of detection.

To evaluate the effects of additive type and concentration on initial biodistribution in mice, additive-free and 10 or 20% additive-containing DOX sheets were applied to the subcutaneous tissues of ICR mice. One day after application, DOX concentrations in the subcutaneous tissues at the application site, plasma, and major organs were measured. As shown in Figure 2, DOX was selectively distributed in the subcutaneous tissues at the application site of the sheet, and DOX concentrations in the spleen, kidneys, and plasma were significantly lower than those in the subcutaneous tissues with additive-free DOX sheets (Figure 2a; *p* < 0.05; Cohen’s *d* range = 6.45–9.32; Supplementary Table S3). Compared to the additive-free DOX sheets, additive-containing DOX sheets exhibited higher mean DOX concentration at the application site 1 day after application. Several additive-containing DOX sheets significantly increased the drug concentration at the application site compared to the additive-free DOX sheets (10% PVP-DOX sheets [*p* < 0.0001, Cohen’s *d* = 6.55]; 20% PVP-DOX sheets [*p* < 0.001, Cohen’s *d* = 3.51]; Figure 2b–d; Supplementary Table S3). In contrast, for plasma and all other analyzed organs (kidneys, liver, and spleen), no significant differences in DOX concentrations were observed between the additive-free and additive-containing DOX sheets. Notably, DOX concentration in the heart was below the detection limit in all groups.

### 3.3. In Vivo Biodistribution of DOX 14 Days After Application of DOX-Loaded Sheets to the Tumor Site

**Figure 3:**
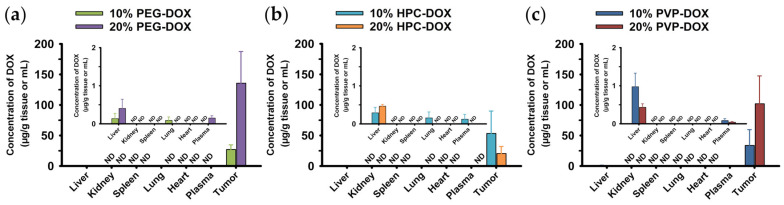
*In vivo* biodistribution of DOX 14 d after application of the following DOX-loaded sheets to the tumor site of Hepa1-6 tumor-bearing mice: (**a**) 10 and 20% PEG-DOX sheets, (**b**) 10 and 20% HPC-DOX sheets, and (**c**) 10 and 20% PVP-DOX sheets. Insets show DOX concentrations in the normal organs and plasma. Each bar represents the mean ± SEM (*n* = 3). ND indicates that all three samples from each organ type of each mouse were below the limit of detection.

After determining that the additive-containing sheets exhibited diverse release profiles and significantly high concentrations at the application site in some cases, these sheets were used for subsequent studies. Additive-containing DOX-loaded sheets were applied for 14 d to the tumor site in C57BL/6J mice generated via subcutaneous injection of Hepa1-6 cells, and DOX distribution in the tumor tissues, normal organs, and plasma was assessed. As shown in Figure 3, sheets containing 10 and 20% PEG, HPC, and PVP tended to exhibit higher DOX distribution in the tumor site than in the other organs. DOX concentrations in most normal organs and plasma were below the detection limit, with some distribution observed in the liver. Among the additive-containing sheets, mean DOX concentration in the tumor tissues was numerically highest in the 20% PEG-DOX sheet group and numerically lowest in the 20% HPC-DOX sheet group, with an approximately 7-fold difference between the two groups (Cohen’s *d* = 1.77).

### 3.4. Relative Body Weight Changes

**Figure 4:**
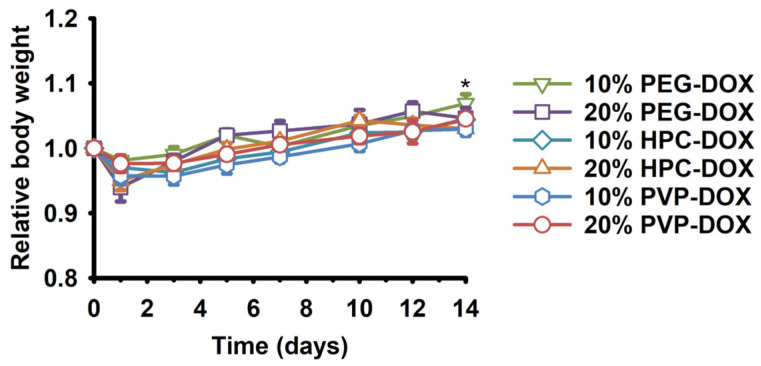
Relative body weight changes in Hepa1-6 tumor-bearing mice treated with the additive-containing DOX-loaded sheets. Each point represents the mean ± SEM (*n* = 3–6). Asterisks indicate significant differences (* *p* < 0.05) from day 0 in the 10% PEG-DOX treatment group, as determined via *t*-tests followed by Bonferroni correction for multiple comparisons.

Body weights of mice treated with the additive-containing DOX-loaded sheets were measured for 14 d. Relative body weight changes in mice after sheet application to subcutaneous tumors are shown in Figure 4. Notably, no significant changes in body weight were observed in the additive-containing sheet groups, except the 10% PEG-DOX group, between day 0 and each subsequent time point. However, body weight was significantly higher on day 14 than on day 0 in the 10% PEG-DOX group (*p* < 0.05, Cohen’s *d* = 1.97, 95% CI [0.03, 0.11]).

### 3.5. In Vivo Antitumor Effects

**Figure 5:**
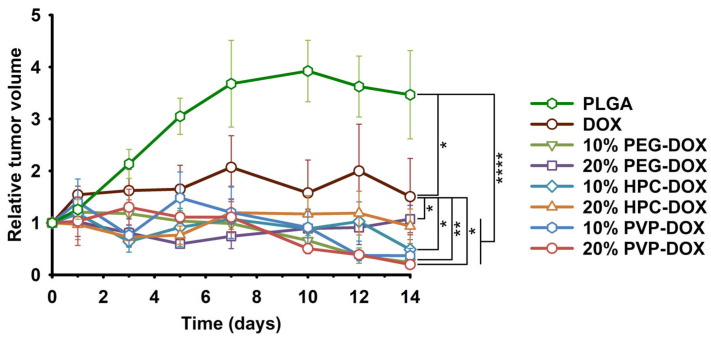
*In vivo* antitumor effects of poly(lactic-*co*-glycolic acid) (PLGA), additive-free DOX, and DOX-loaded sheets containing 10 and 20% PEG, HPC, and PVP on Hepa1-6 tumor-bearing mice over time after application to the tumor site. Each point represents the mean ± SEM (*n* = 3–6). Asterisks indicate the significant differences between two groups, as determined via pairwise Welch’s *t*-tests followed by Bonferroni correction (* *p* < 0.05, ** *p* < 0.01, and **** *p* < 0.0001).

Next, *in vivo* antitumor effects of the additive-containing DOX-loaded sheets were confirmed using Hepa1-6 subcutaneous tumor-bearing C57BL/6J mice. Relative tumor volume changes in the additive-containing DOX-loaded sheet groups compared to those in the DOX-free and additive-free cover PLGA sheet and additive-free DOX-loaded sheet groups as controls are shown in Figure 5. Compared to that in the additive-free DOX-loaded sheet group, tumor growth was significantly suppressed in the additive-containing sheet groups, except the 20% HPC-DOX and 10% PVP-DOX groups (*p* < 0.05; Cohen’s *d* range = 0.88–1.02; Supplementary Table S4).

**Figure 6:**
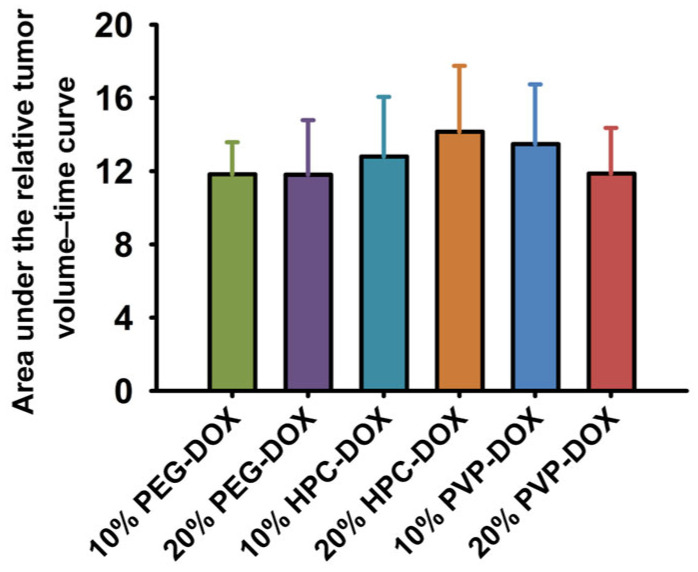
Comparison of the area under the relative tumor volume–time curve among the additive-containing DOX sheet groups, calculated using the data shown in Figure 5. Data are represented as the mean ± SEM (*n* = 3–6). One-way AVOVA revealed no statistically significant differences among the additive-containing DOX sheet groups.

For the comprehensive and quantitative comparison of the overall tumor growth curve in the additive-containing sheet groups, the mean area under the relative tumor volume–time curve over 14 d was calculated for each group (Figure 6). Notably, one-way ANOVA revealed no statistically significant differences among the six additive-containing sheet groups.

### 3.6. Associations Between Antitumor Effects and Tumor Concentrations

**Figure 7:**
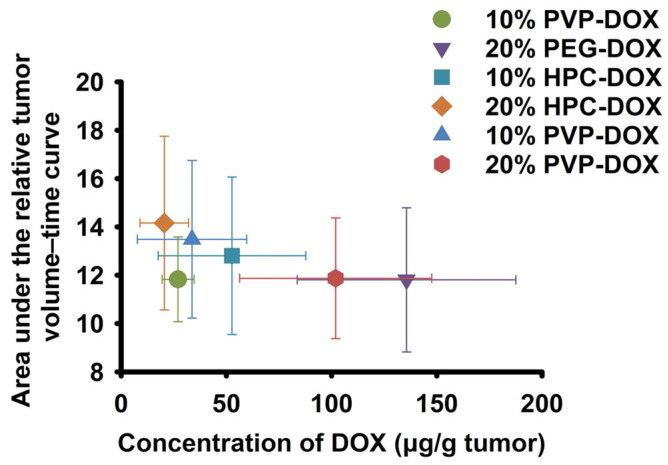
Relationship between mean antitumor effects and mean tumor tissue concentrations of DOX-loaded sheets containing 10 and 20% PEG, HPC, and PVP on Hepa1-6 tumor-bearing mice over time after application to the tumor site. Each point represents the mean ± SEM (*n* = 3–6 for antitumor effects; *n* = 3 for tumor tissue concentrations). In the 10% PEG-DOX, 20% PEG-DOX, and 10% HPC-DOX sheet groups, the numbers of mice used to calculate the mean tumor concentration on day 14 and mean area under the relative tumor volume–time curve over 14 d differed due to differences in data availability.

As the overall antitumor effects, quantified by the mean area under the relative tumor volume–time curve, were statistically indistinguishable among the additive-containing DOX sheets, we examined whether local tumor drug concentrations are associated with these effects. The association between the mean area under the relative tumor volume–time curve and mean tumor DOX concentration was examined. As shown in Figure 7, the scatter plot indicated that the mean area under the relative tumor volume–time curve tended to decrease with increasing mean DOX concentration after 14 d in all tested groups, except the 10% PEG-DOX group. Specifically, 20% PEG-DOX sheet group exhibited the highest mean DOX concentration in the tumor tissues after 14 days, along with a low mean area under the relative tumor volume–time curve, suggesting enhanced antitumor effects. Conversely, 20% HPC-DOX sheet group exhibited the lowest mean DOX concentration in the tumor tissues after 14 d, along with a high mean area under the relative tumor volume–time curve, suggesting reduced antitumor effects.

Based on these findings, two formulations with contrasting effects, 20% HPC-DOX and 20% PEG-DOX, were investigated. Initial biodistribution was assessed by quantifying DOX concentrations in the tumor tissues, major organs, and plasma 1 day after application (Table 2), and tumor concentrations were compared with those measured 14 d after application (Figure 3).

In the 20% HPC-DOX group, mean tumor DOX concentration after 14 d was approximately 4% of that after 1 d, changing from 496 ± 324 µg/g tissue to 20.5 ± 11.5 µg/g tissue, although this difference was not statistically significant (Cohen’s *d* = 1.20; 95% CI [−918, 1869]). In contrast, in the 20% PEG-DOX group, mean tumor DOX concentration after 14 d was approximately 70% of that after 1 day, changing from 190 ± 108 µg/g tissue to 136 ± 51.9 µg/g tissue, with no significant difference (Cohen’s *d* = 0.37; 95% CI [−279, 388]).

## 4. Discussion

Local chemotherapy is important to increase the drug concentration at the target site, improving the therapeutic efficacy and reducing the systemic side effects of conventional chemotherapy approaches, such as oral or parenteral drug administration [22]. To deliver drugs directly to tumors, we developed sheets loaded with DOX, an anticancer drug used to treat several solid tumors [15]. After application, the sheets were expected to gradually release DOX locally and then degrade; therefore, PLGA, which is used for sustained delivery systems [23] and is biodegradable [24], was selected for this study.

As the overall DOX release profile from the PLGA polymer was low, other formulation conditions were explored by evaluating various types of sheets with enhanced release profiles. The use of water-soluble polymer additives (PEG, HPC, and PVP) in this study was consistent with previous reports of additives modulating the drug release profiles of PLGA matrices [25,26]. These polymers are biocompatible, soluble in various organic solvents, and exhibit diverse industrial applications, including controlled drug delivery [27,28,29,30,31,32,33,34]. In this study, we developed six types of DOX-loaded sheets with different additives and concentrations and determined the factors influencing their efficacy by evaluating their *in vitro* release profiles, *in vivo* biodistribution, and antitumor effects.

*In vitro* drug release tests showed that the sheets released DOX gradually after an initial burst. Initial release occurs when the drug molecules on or near the surface dissolve and are released into the medium [35]. Release of surface drugs with low solubility into the polymer matrices is a possible contributing factor to the burst effect [36]. The initial release from the sheets was possibly due to the release of DOX molecules from the surface of the sheets near the aqueous medium. Moreover, DOX molecules on the sheet surface with low solubility in polymer matrices probably further promoted the initial burst. The overall mean DOX release increased in the presence of additives. Drug content affects drug release in progesterone-containing PLGA microspheres [37]. High DOX release from the additive-containing sheets was possibly because the additives influenced the initial DOX loading of the sheets. Moreover, inclusion of additives possibly increased the phase separation and dissolution of additive-rich domains in the release medium. Notably, cumulative amount of DOX released from the 10% PEG-DOX sheets during the initial phase was lower than that released from the additive-free DOX sheets. PEG possibly acts as a plasticizer for PLGA, lowering its glass-transition temperature [38]. The polymer possibly becomes rubbery, causing drug diffusion through the polymer matrix [38,39,40]. Further studies are necessary to understanding the drug release mechanisms from the sheets, including the surface and cross-sectional morphology, glass transition temperature, and initial drug loading of the sheets. Our drug release study suggests that the inclusion of additives in the sheets enhances DOX release, with the additive type and concentration potentially affecting the release profiles of the sheets.

Biodistribution studies revealed that DOX concentrations remained higher at the application site than at the other sites in ICR mice. This suggests that the sheets inhibit DOX distribution in systemic tissues by locally releasing DOX. DOX concentrations were below the detection limit in some organs, including the heart. This suppression of DOX distribution to the heart is important because myocardial damage is a serious adverse effect of DOX. Compared to the additive-free DOX sheets, DOX sheets containing 10 and 20% additives exhibited higher DOX concentrations at the application site. A previous study reported that the liver concentration of 5-fluorouracil at the application site is maintained relative to that at other live sites, particularly in the presence of polyvinyl alcohol [41]. Therefore, inclusion of additives in the sheets is important to increase the DOX concentration at the application site.

Liver surface is used as an administration route to investigate the absorption of various compounds, including DOX [42]. Therefore, this study evaluated the potential of DOX-loaded sheets to treat liver cancer using Hepa1-6 tumor-bearing mice. Biodistribution studies were performed after applying additive-containing sheets to the subcutaneous tumors in C57BL/6J mice for 14 d. Notably, DOX was distributed in the tumor even 14 days after sheet application. DOX concentrations in normal organs and plasma were undetectable, indicating that DOX was possibly eliminated via the blood circulation. All types of additive-containing sheets exhibited low DOX concentrations in the liver, possibly because the liver is a major site of DOX metabolism [43]. These results highlight the potential of direct DOX-loaded sheet application to tumor sites for localized therapy. In this study, no obvious body weight loss was observed (Figure 4). Previous studies have reported considerable weight loss under DOX-induced toxicity [44,45].

Antitumor effects of the additive-containing DOX-loaded sheets, other than the 20%-HPC-DOX and 10% PVP-DOX sheets, were significantly more potent than those of the additive-free DOX sheets (Figure 5), suggesting that the inclusion of additives in the sheets improved their antitumor effectiveness. The area under the curve test was used to determine the factors contributing to the antitumor effects of the additive-containing DOX sheets. Although tumor growth measurements vary across time points, hindering direct comparison, area under the curve provides a comprehensive solution by quantifying the entire tumor growth curve in a single number [46]. Notably, the area under the curve indicated that the overall antitumor effects were statistically indistinguishable among the six additive-containing sheets (Figure 6). This finding suggests that equivalent overall antitumor effects do not always correspond to equivalent tumor concentrations. Moreover, additive-containing DOX-loaded sheets exhibited diverse release profiles and different tissue concentrations at the application site, suggesting that the underlying therapeutic mechanisms involve a complex interplay between the overall antitumor effects and drug concentrations at the tumor site. To verify this, the relationship between the mean tumor concentration after 14 days and mean area under the relative tumor volume–time curve over 14 days was examined. Although statistical correlation analysis was not performed due to a discrepancy in the number of samples for the two variables, the data indicated an inverse trend between the mean tumor concentrations and mean area under the relative tumor volume–time curve (Figure 7). This result suggests an association between the drug concentration at the tumor site and overall antitumor effect.

The contrasting tumor concentration profiles of the 20% PEG-DOX and 20% HPC-DOX sheets may explain the mechanism underlying their overall antitumor effects. Although the data after 1 and 14 d were obtained from different mice and sheet batches, mean concentration in the 20% HPC-DOX sheet group after 14 d was approximately 4% compared to that after 1 day, whereas that in the 20% PEG-DOX sheet group after 14 days was approximately 70% of that after 1 day; however, the differences were not statistically significant. These distinct retention profiles suggest different modes of therapeutic action: the 20% HPC-DOX sheets likely exerted their effect primarily through a high initial concentration followed by a rapid decline, whereas the 20% PEG-DOX sheets likely achieved comparable antitumor effect through sustained tumor exposure. This addresses the critical question regarding the concentration-effect relationship: our findings suggest that sustained tumor exposure, rather than just peak or final concentrations, may be a factor contributing to the antitumor effects, offering a strategy to achieve comparable efficacy without relying on extremely high peak concentrations.

One limitation of this study is the heterogeneous distribution of DOX in the sheets. The formation of DOX aggregates contributed to the non-uniform loading of DOX in the sheets. They were formed randomly within the diffusion cell, making it difficult to control the formation of DOX concentration gradients and aggregates in the sheets. Therefore, the sheets must eventually be cut out, and variations in initial drug loading due to the non-uniform distribution of DOX in the sheets must be carefully considered. Thus, comparative findings between different formulations warrant careful interpretation, as the possibility of loading variability cannot be excluded, even among sheets with nominally identical formulations. Additional studies are necessary to further assess the initial drug loading. Fabricating sheets with a uniform DOX concentration can facilitate more in-depth analysis of the initial drug loading. Another limitation is that histopathological confirmation of necrosis/apoptosis, biochemical toxicity markers, and hemolysis testing were not performed. Future studies should include these comprehensive safety assessments, as well as mechanistic analyses investigating how release kinetics relate to intratumoral pharmacodynamics, to more fully evaluate the biocompatibility and therapeutic potential of the DOX-loaded two-layered sheets.

In summary, we developed additive-containing DOX-loaded sheets for direct application to tumor surfaces. These sheets exhibited antitumor effects comparable to or superior to those of additive-free DOX sheets, showing great therapeutic potential. Therefore, two-layered DOX-loaded sheets can potentially be attached to the surface of cancerous organs in the abdominal cavity, such as the liver and kidneys, for postoperative adjuvant chemotherapy. Our results can contribute to the development of effective two-layered DOX-loaded sheets for improved chemotherapy outcomes.

## 5. Conclusions

In conclusion, this study established two-layered DOX-loaded sheets as a promising platform for localized chemotherapy. Our developed additive-containing DOX-loaded sheets exhibit great therapeutic potential for direct tumor application. Our findings suggest that their antitumor mechanisms possibly rely on the tumor exposure profile rather than the absolute drug concentration at the tumor site. Collectively, our findings provide valuable guidance for the development of locally administrable DOX-loaded sheets.

## Figures and Tables

**Table 1:** Composition of the polymer and drug mixtures used to prepare the DOX-loaded two-layered sheets.

Formulation	Drug-Loaded Layers					Drug-Free Layers
	PLGA (µL)	DOX (µL)	Additive	Additive Amount (µL)	Additive Content (*w*/*w*, %)	PLGA (µL)
PLGA	–	–	–	–	–	400
DOX	800	9	–	0	0	400
10% PEG-DOX	800	9	PEG	899	10	400
20% PEG-DOX	800	9	PEG	2022	20	400
10% HPC-DOX	800	9	HPC	899	10	400
20% HPC-DOX	800	9	HPC	2022	20	400
10% PVP-DOX	800	9	PVP	899	10	400
20% PVP-DOX	800	9	PVP	2022	20	400

PLGA solution: 10% (*w*/*v*) in acetone. Additive solution: 1% (*w*/*v*) in acetone. DOX solution: 100 mg/mL in dimethyl sulfoxide. The total nominal DOX loading is 2 µL (100 mg/mL) in all DOX-loaded sheets (excluding PLGA sheets). The actual drug content in the final cut-out samples may vary due to the formation of aggregates, as discussed in the Limitations section. All sheets were prepared using a cylindrical glass diffusion cell (inner diameter: 18 mm) and cut into semicircles (diameter: 12 mm) after air-drying. Abbreviations: DOX, doxorubicin; PLGA, poly(lactic-*co*-glycolic acid); PEG, polyethylene glycol; HPC, hydroxypropyl cellulose; PVP, Polyvinylpyrrolidone.

**Table 2:** DOX concentrations in tumor, normal organ, and plasma samples 1 day after application of 20% PEG-DOX or 20% HPC-DOX sheets. Data are represented as the mean ± standard error of the mean (SEM) (*n* = 3).

Samples	Unit	20% PEG-DOX	20% HPC-DOX
Liver	µg/g tissue	3.47 ± 0.270	3.12 ± 0.908
Kidney	µg/g tissue	1.83 ± 0.267	3.23 ± 0.671
Spleen	µg/g tissue	0.880 ± 0.880	4.19 ± 1.01
Lung	µg/g tissue	1.15 ± 0.232	2.28 ± 0.422
Heart	µg/g tissue	0.493 ± 0.256	1.67 ± 0.433
Plasma	µg/mL	0.0962 ± 0.0114	0.119 ± 0.0631
Tumor	µg/g tissue	190 ± 108	496 ± 324

## Data Availability

Raw data supporting the conclusions of this study are available upon request from the authors.

## Supplementary Materials

The following supporting information can be downloaded at: https://www.mdpi.com/article/10.3390/pharmaceutics17121565/s1, Table S1. Thickness of DOX-loaded two-layered sheets; Table S2. Statistical analysis of in vitro cumulative DOX release profiles; Table S3. Statistical analysis of DOX concentrations in plasma and tissues; Table S4. Statistical analysis of antitumor effects.

## Abbreviations

The following abbreviations are used in this manuscript:

DOX	Doxorubicin
PLGA	Poly(lactic-*co*-glycolic acid)
PEG	Polyethylene glycol
HPC	Hydroxypropyl cellulose
PVP	Polyvinylpyrrolidone
