# Supplementary material for: Development and Antitumor Evaluation of Doxorubicin-Loaded Two-Layered Sheets for Local Chemotherapy via Direct Drug Application to the Tumor Surface
Source: Pharmaceutics. 2025 Dec 4;17(12):1565. doi: 10.3390/pharmaceutics17121565 (PMC12736872; doi:10.3390/pharmaceutics17121565)
Supplement: Supplementary file 1 [file pharmaceutics-17-01565-s001.zip › pharmaceutics-3980385-supplementary.pdf]

Supplementary Table S1. Thickness of DOX-loaded two-layered sheets. Data are represented as the mean ± standard deviation (SD) of three sheets (n=3). Measurements were performed using a digital caliper with a resolution of 0.1 mm.

Formulation	Thickness (mm)
DOX	0.2 ± 0.1
10% PEG-DOX	0.2 ± 0.1
20% PEG-DOX	0.3 ± 0.1
10% HPC-DOX	0.3 ± 0.0
20% HPC-DOX	0.2 ± 0.1
10% PVP-DOX	0.2 ± 0.0
20% PVP-DOX	0.3 ± 0.1

Supplementary Table S2. Statistical analysis of *in vitro* cumulative DOX release profiles: Pairwise comparisons between additive-free and additive-containing DOX sheets.

Formulation (vs DOX)	Mean Difference (μg)	95% CI [Lower, Upper]	Cohen's *d*	Adjusted *p*-value
10% PEG-DOX	9.78	[3.71, 15.85]	0.84	0.050
20% PEG-DOX	-93.74	[-112.37, -75.12]	2.64	< 0.0001
10% HPC-DOX	-58.03	[-78.52, -37.55]	1.49	< 0.0001
20% HPC-DOX	-57.69	[-75.13, -40.26]	1.73	< 0.0001
10% PVP-DOX	-41.01	[-57.02, -24.99]	1.34	< 0.001
20% PVP-DOX	-136.63	[-165.00, -108.25]	2.53	< 0.0001

Data were analyzed using Welch's *t*-test followed by Bonferroni correction for multiple comparisons. CI: Confidence Interval. Effect sizes were calculated as Cohen's *d*.

Supplementary Table S3. Statistical analysis of DOX concentrations in plasma and tissues. (A) Pairwise comparisons between the application site (subcutaneous tissue) and other tissues for each formulation (Paired *t*-test with Bonferroni correction).

Formulation	Tissue (vs subcutaneous tissue)	Mean Difference (μg/g tissue or μg/mL)	95% CI [Lower, Upper]	Cohen's *d*	Adjusted *p*-value
DOX	Liver	-3.59	[-5.36, -1.82]	5.04	0.077
DOX	Kidney	-4.68	[-5.93, -3.43]	9.32	0.023
DOX	Spleen	-4.74	[-6.56, -2.91]	6.45	0.048
DOX	Lung	-4.87	[-6.92, -2.83]	5.91	0.056
DOX	Heart	-4.87	[-6.92, -2.83]	5.91	0.056
DOX	Plasma	-4.75	[-6.54, -2.97]	6.61	0.045
10% PEG-DOX	Liver	-18.17	[-36.49, 0.16]	2.46	0.305
10% PEG-DOX	Kidney	-18.43	[-37.05, 0.18]	2.46	0.306
10% PEG-DOX	Spleen	-18.08	[-36.73, 0.58]	2.41	0.318
10% PEG-DOX	Lung	-18.27	[-36.82, 0.28]	2.45	0.309
10% PEG-DOX	Heart	-18.80	[-37.80, 0.20]	2.46	0.306
10% PEG-DOX	Plasma	-18.72	[-37.60, 0.15]	2.46	0.305
20% PEG-DOX	Liver	-15.92	[-24.50, -7.34]	4.61	0.092
20% PEG-DOX	Kidney	-17.17	[-28.16, -6.17]	3.88	0.129
20% PEG-DOX	Spleen	-16.82	[-27.10, -6.54]	4.06	0.118
20% PEG-DOX	Lung	-17.18	[-27.75, -6.62]	4.04	0.119

20% PEG-DOX	Heart	-17.36	[-27.74, -6.98]	4.15	0.113
20% PEG-DOX	Plasma	-17.28	[-27.61, -6.95]	4.15	0.113
10% HPC-DOX	Liver	-6.99	[-17.14, 3.17]	1.71	0.586
10% HPC-DOX	Kidney	-7.25	[-16.30, 1.80]	1.99	0.449
10% HPC-DOX	Spleen	-6.92	[-15.40, 1.56]	2.03	0.435
10% HPC-DOX	Heart	-7.25	[-16.30, 1.80]	1.99	0.449
10% HPC-DOX	Lung	-7.25	[-16.30, 1.80]	1.99	0.449
10% HPC-DOX	Plasma	-7.17	[-16.23, 1.88]	1.97	0.458
20% HPC-DOX	Liver	-9.05	[-20.89, 2.78]	1.90	0.487
20% HPC-DOX	Kidney	-9.62	[-22.45, 3.21]	1.86	0.504
20% HPC-DOX	Spleen	-9.40	[-21.90, 3.09]	1.87	0.501
20% HPC-DOX	Heart	-9.85	[-23.56, 3.85]	1.79	0.543
20% HPC-DOX	Lung	-9.68	[-22.70, 3.35]	1.85	0.513
20% HPC-DOX	Plasma	-9.76	[-23.35, 3.82]	1.79	0.543
10% PVP-DOX	Liver	-81.21	[-276.20, 113.78]	1.03	1.000
10% PVP-DOX	Kidney	-81.63	[-275.80, 112.54]	1.04	1.000
10% PVP-DOX	Spleen	-80.55	[-273.80, 112.70]	1.04	1.000
10% PVP-DOX	Lung	-81.67	[-274.87, 111.54]	1.05	1.000
10% PVP-DOX	Heart	-83.06	[-278.03, 111.92]	1.06	1.000
10% PVP-DOX	Plasma	-82.94	[-277.95, 112.06]	1.06	1.000
20% PVP-DOX	Liver	-45.02	[-108.17, 18.13]	1.77	0.551
20% PVP-DOX	Kidney	-45.54	[-108.97, 17.90]	1.78	0.545
20% PVP-DOX	Spleen	-45.29	[-106.67, 16.09]	1.83	0.519
20% PVP-DOX	Lung	-46.46	[-110.34, 17.43]	1.81	0.533
20% PVP-DOX	Plasma	-46.77	[-111.84, 18.30]	1.79	0.544
20% PVP-DOX	Heart	-46.84	[-112.18, 18.50]	1.78	0.546

(B) Pairwise comparisons between additive-free and additive-containing DOX sheets for each tissue (Dunnett's test).

**Tissue**	**Formulation (vs DOX)**	**Mean Difference (μg/g tissue or μg/mL)**	**95% CI [Lower, Upper]**	**Cohen's *d***	**Adjusted *p*-value**
Liver	10% PEG-DOX	-0.65	[-26.27, 24.96]	0.05	1.000
Liver	20% PEG-DOX	0.16	[-25.46, 25.77]	0.01	1.000
Liver	10% HPC-DOX	-1.02	[-26.63, 24.59]	0.09	1.000
Liver	20% HPC-DOX	-0.48	[-26.10, 25.13]	0.04	1.000
Liver	10% PVP-DOX	0.57	[-25.05, 26.18]	0.05	1.000
Liver	20% PVP-DOX	0.54	[-25.08, 26.15]	0.05	1.000
Kidney	10% PEG-DOX	0.17	[-25.44, 25.79]	0.01	1.000
Kidney	20% PEG-DOX	-0.00	[-25.62, 25.61]	0.00	1.000
Kidney	10% HPC-DOX	-0.19	[-25.81, 25.42]	0.02	1.000
Kidney	20% HPC-DOX	0.04	[-25.58, 25.65]	0.00	1.000
Kidney	10% PVP-DOX	1.23	[-24.38, 26.85]	0.10	0.999
Kidney	20% PVP-DOX	1.11	[-24.50, 26.73]	0.09	1.000
Spleen	10% PEG-DOX	0.59	[-25.03, 26.20]	0.05	1.000
Spleen	20% PEG-DOX	0.40	[-25.22, 26.01]	0.03	1.000
Spleen	10% HPC-DOX	0.20	[-25.42, 25.81]	0.02	1.000
Spleen	20% HPC-DOX	0.32	[-25.30, 25.92]	0.03	1.000
Spleen	10% PVP-DOX	2.38	[-23.24, 27.99]	0.20	0.997
Spleen	20% PVP-DOX	1.42	[-24.20, 27.03]	0.12	0.999
Lung	10% PEG-DOX	0.53	[-25.08, 26.15]	0.04	1.000
Lung	20% PEG-DOX	0.17	[-25.44, 25.79]	0.01	1.000

Lung	10% HPC-DOX	0.00	[-25.61, 25.61]	0.00	1.000
Lung	20% HPC-DOX	0.18	[-25.44, 25.79]	0.02	1.000
Lung	10% PVP-DOX	1.38	[-24.22, 27.01]	0.12	0.999
Lung	20% PVP-DOX	0.38	[-25.23, 26.00]	0.03	1.000
Heart	10% PEG-DOX	0.00	[-25.61, 25.61]	0.00	1.000
Heart	20% PEG-DOX	0.00	[-25.61, 25.61]	0.00	1.000
Heart	10% HPC-DOX	0.00	[-25.61, 25.61]	0.00	1.000
Heart	20% HPC-DOX	0.00	[-25.61, 25.61]	0.00	1.000
Heart	10% PVP-DOX	0.00	[-25.61, 25.61]	0.00	1.000
Heart	20% PVP-DOX	0.00	[-25.61, 25.61]	0.00	1.000
Plasma	10% PEG-DOX	-0.04	[-25.66, 25.57]	0.00	1.000
Plasma	20% PEG-DOX	-0.04	[-25.66, 25.57]	0.00	1.000
Plasma	10% HPC-DOX	-0.04	[-25.66, 25.57]	0.00	1.000
Plasma	20% HPC-DOX	-0.03	[-25.64, 25.59]	0.00	1.000
Plasma	10% PVP-DOX	0.00	[-25.62, 25.61]	0.00	1.000
Plasma	20% PVP-DOX	-0.05	[-25.66, 25.57]	0.00	1.000
Subcutaneous	10% PEG-DOX	13.92	[-11.69, 39.54]	1.17	0.511
Subcutaneous	20% PEG-DOX	12.48	[-13.13, 38.10]	1.05	0.605
Subcutaneous	10% HPC-DOX	2.38	[-23.24, 27.99]	0.20	0.996
Subcutaneous	20% HPC-DOX	4.98	[-20.63, 30.60]	0.42	0.963
Subcutaneous	10% PVP-DOX	78.18	[57.57, 103.80]	6.55	<0.0001
Subcutaneous	20% PVP-DOX	41.97	[16.35, 67.58]	3.51	<0.001

Note: Data were analyzed using (A) Dunnett's test following two-way ANOVA for comparisons against the control group (DOX sheets), and (B) paired *t*-test with Bonferroni correction for comparisons within each formulation against the application site (subcutaneous tissue). CI: Confidence Interval. Effect sizes were calculated as Cohen's *d*.

Supplementary Table S4. Statistical analysis of antitumor effects (relative tumor volume). Pairwise comparisons between the additive-free DOX sheet and other formulations (Welch's *t*-test with Bonferroni correction).

Formulation (vs DOX)	Mean Difference (Relative tumor volume)	95% CI [Lower, Upper]	Cohen's *d*	Adjusted *p*-value
10% PEG-DOX	-0.79	[-1.19, -0.39]	1.02	< 0.01
20% PEG-DOX	-0.74	[-1.15, -0.33]	0.92	< 0.05
10% HPC-DOX	-0.72	[-1.13, -0.31]	0.88	< 0.05
20% HPC-DOX	-0.63	[-1.05, -0.21]	0.74	0.119
10% PVP-DOX	-0.68	[-1.13, -0.23]	0.77	0.100
20% PVP-DOX	-0.79	[-1.22, -0.36]	0.92	< 0.05
PLGA	-1.15	[-1.82, -0.48]	0.97	< 0.05

Note: CI: Confidence Interval. Effect sizes were calculated as Cohen's *d*.
